# A Law of Cure

**Published:** 1869-07

**Authors:** 


### A LAW OF CURE.

BY P. H. H.

If you were to go into the city of New York with chronic rheumatism, and enquire where you could be cured, you might be recommended by different parties to try the Turkish bath, or the Russian Bath, or the Movement cure, or the Lifting cure, or the Pneumatic cure, or electricity. You would ask with amazement, What shall I do? How is it, that all these modes of treatment acquire currency and credit, and keep an existence in this great city?

"Whether but one member suffer all the members suffer with it." If a man has rheumatism in his joints he is rheumatism-*sick*—all over. If a man has dyspepsia in his stomach he has it in his brain, muscles, temper; he is *dyspepsia-sick*. If a man has the tooth-ache he is tooth-ache-sick—can't attend to business. If a man runs a nail into the bottom of his foot, his whole nervous system is thrown into a most violent irritation—may be tetanic *spasm*. If a man has just a hard cold, he is "half-sick", all over. Don't you see that a single organ sick or hurt makes all the body sick? A single nerve wounded sends a thrill of pain and irritation through the whole body.

Now what is the rest of that scripture—"or one member be honored, all the members rejoice with it." Aye, now we have it. The man who gives baths has studied the skin. He has learned that if the skin is chilly a man is all uncomfortable. If the skin is dry and feverish, or if it is the seat of any irritation, he is still more so. He has learned, moreover, that any suspension of healthy action in the skin interferes with the elimination of waste and disease-causing matters.

Now, he says, I will intensify the conditions of health in the skin; I will give my patient a bath daily; I will rub him and shampoo him thoroughly. Don't I know as much about a *man's* health as a groom does about a *horse's*? I will give him plenty of exercise to send the blood well into the skin. I will keep his skin in a happy sense of comfort by just the right amount of clothing, and the thing is done. I'll cure my man. And he *does*; and the reason is, if the health of the skin is exalted, every vital function is improved.

So the man who uses the movement-cure, and the man who prescribes the lifting-cure, acts upon the muscular system—which is more than half the weight of a strong man—and acts to produce a high state of health in this, and while doing so every part of the body and mind feels the sympathy from this good influence, and the end is established health.

So the man who "houses" a lazy liver, with a dose of *electricity*, helps the whole body to comfort and health. Now, if each of the doctors always got the cases to which his speciality was best adapted, and did not get any of the others, all would be well; but there is the rub. Well, go to a doctor, who understands and uses all these means, and modifies and accepts any or all of these means to his various cases of chronic disease.

Now I have answered my question, and clinched into my memory St. Paul's saying, that "whether one member suffer all, and the members suffer with it; or one member be honored, all the members rejoice with it."

